# Mosquito Larvicidal Activity of the Essential Oils of *Erechtites* Species Growing Wild in Vietnam

**DOI:** 10.3390/insects10020047

**Published:** 2019-02-03

**Authors:** Nguyen Huy Hung, Prabodh Satyal, Ho Viet Hieu, Nguyen Thi Hong Chuong, Do Ngoc Dai, Le Thi Huong, Thieu Anh Tai, William N. Setzer

**Affiliations:** 1 Center for Advanced Chemistry, Institute of Research and Development, Duy Tan University,03 Quang Trung, Da Nang 50000, Vietnam; huyhung2602@gmail.com (N.H.H.); hongchuong1991@gmail.com (N.T.H.C.); 2 Aromatic Plant Research Center, 230 N 1200 E, Suite 102, Lehi, UT 84043, USA; psatyal@aromaticplant.org; 3 Parasitology and Entomology Unit, Department of Medicine, Duy Tan University, 03 Quang Trung, Da Nang 50000, Vietnam; 4 Faculty of Agriculture, Forestry and Fishery, Nghe An Economics University, Vinh City 43000, Nghe An Province, Vietnam; daidn23@gmail.com; 5 School of Natural Science Education, Vinh University, 182 Le Duan, Vinh City 43000, Nghệ An Province, Vietnam; lehuong223@gmail.com; 6 Department of Pharmacy, Duy Tan University, 03–Quang Trung, Da Nang 50000, Vietnam; anhtai0808qn@gmail.com; 7 Department of Chemistry, University of Alabama in Huntsville, Huntsville, AL 35899, USA

**Keywords:** *Erechtites hieraciifolius*, *Erechtites valerianifolius*, chemical composition, α-pinene, limonene, myrcene, β-caryophyllene, caryophyllene oxide

## Abstract

Mosquito-borne infections are a constant problem in Vietnam, and mosquito vector control is a primary approach to control these infections. Essential oils represent environmentally friendly alternatives to synthetic pesticides for mosquito control. The essential oils of two weedy species in Vietnam, *Erechtites hieraciifolius* and *E. valerianifolius*, have been obtained by hydrodistillation and analyzed by gas chromatography–mass spectrometry. The essential oils have been screened for mosquito larvicidal activity against *Aedes albopictus*, *Ae. aegypti*, and *Culex quinquefasciatus*. The essential oil from the aerial parts of *E. hieraciifolius* was rich in α-pinene (14.5%), limonene (21.4%), and caryophyllene oxide (15.1%), while *E. valerianifolius* essential oil was dominated by myrcene (47.8%) and α-pinene (30.2%). Both essential oils showed good larvicidal activity against *Ae. albopictus* (24-h LC_50_ 10.5 and 5.8 μg/mL, respectively) and *Ae. aegypti* (24-h LC_50_ 10.6 and 12.5 μg/mL, respectively). The essential oil of *E. valerianifolius* also showed good activity against *Cx. quinquefasciatus* larvae (24-h LC_50_ = 40.7 μg/mL). Thus, *Erechtites* essential oils may serve as low-cost vector control agents for mosquito-borne infections.

## 1. Introduction

*Aedes aegypti* (L.) and *Ae. albopictus* (Skuse) (Diptera: Culicidae) are important vectors of arboviral infections, including yellow fever, dengue, Zika, and chikungunya [1,2,3]. Vietnam is classified as a hyperendemic dengue country, with all four dengue serotypes present throughout the year [4]. In the last half century, dengue fever epidemics have increased in frequency, corresponding to a median annual incidence of 232 cases per 100,000 people [4]. Furthermore, chikungunya is expected to become a major health threat in Vietnam in the near future [4,5].

Vector control is one of the primary approaches to reduce the spread of arboviral infections. However, current methods for controlling *Aedes* mosquitoes have been largely ineffective [6]. Botanical insecticides in general [7,8] and essential oils in particular [9,10] have emerged as promising, environmentally friendly alternatives to synthetic pesticides for mosquito control.

There are around 12 species of *Erechtites* (Asteraceae), and they are native to North America, West Indies, South America, New Zealand, and Australia [11]. *Erechtites hieraciifolius* (L.) Raf. ex DC. (syn. *Erechtites hieracifolia* (L.) Raf., *Erechtites hieraciifolia* (L.) Raf. ex DC.,) is native to North America, South America, and the West Indies, but it has been introduced to Europe, Hawaii, and Asia [12,13,14,15,16]. *Erechtites valerianifolius* (Wolf) DC. (syn. *Erechtites valerianifolia* (Link ex Wolf) Less. ex DC., *Erechtites valerianaefolia* (Wolf) DC.) is native to Central and South America, but this species has also has been introduced to Asia [13,14,15,17,18].

*Erechtites hieraciifolius* is used traditionally in Venezuela (a plant decoction is used as a bath to reduce fever) and in El Salvador (a decoction is used to treat coughs) [19]. In Bolivia, the Tacana people use an oil extract of *E. hieraciifolius* to treat wounds and pimples [20]. An ethanol extract of *E. hieraciifolius* showed in vitro antileishmanial activity against promastigotes of *Leishmania* (*Leishmania*) *amazonensis* Lainson & Shaw and *L.* (*Viannia*) *braziliensis* Vianna [20]. In North America, *E. hieraciifolius* was previously used to treat hemorrhages, wounds, skin diseases, and as a topical treatment for poison ivy (*Toxicodendron radicans* (L.) Kuntze, Anacardiaceae) and poison sumac (*T. vernix* (L.) Kuntze) rash [21].

As part of our ongoing research on identifying the potential utility of invasive plant species in Vietnam, we have obtained the essential oils from *E. hieraciifolius* and *E. valerianifolius* and have examined their mosquito larvicidal activities. In order to assess the potential environmental impact of using *Erechtites* essential oils as a larvicidal control agent, we have carried out lethality assays on the non-target aquatic species. As far as we are aware, there have been no previous investigations on the larvicidal activities of *Erechtites* essential oils.

## 2. Materials and Methods

### 2.1. Plant Material

Aerial parts of *E. valerianifolius* were harvested from plants growing in Dong Giang district, Quang Nam Province (15°58′9.8″ N, 107°55′4.7″ E; sample Quang Nam), Hoa Vang district, Da Nang city (16°01′0.6″ N, 108°4′25.6″ E;), while aerial parts of *E. hieraciifolius* were harvested from plants growing in Hoa Vang district, Da Nang city (16°2′22.0″ N, 108°3′33.0″ E), in April 2018. The plants were identified by Dr. Do Ngoc Dai, and voucher specimens (LTH127 and LTH128, respectively) have been deposited in the Pedagogical Institute of Science, Vinh University. Fresh plant materials (leaves, stems, and flowers) were kept at room temperature (≈25 °C), and 2 kg samples of each of the plant materials were shredded and hydrodistilled for 4 h using a Clevenger type apparatus.

### 2.2. Gas Chromatographic—Mass Spectral Analysis

Each of the *Erechtites* essential oils was analyzed by gas chromatography–mass spectrometry (GC-MS) using a Shimadzu GCMS-QP2010 Ultra operated in the electron impact (EI) mode (electron energy = 70 eV), scan range = 40–400 atomic mass units, scan rate = 3.0 scans/s, and GC–MS solution software. The GC column was a ZB-5 fused silica capillary column with a (5% phenyl)-polymethylsiloxane stationary phase and a film thickness of 0.25 μm. The carrier gas was helium with a column head pressure of 552 kPa and flow rate of 1.37 mL/min. The injector temperature was 250 °C and the ion source temperature was 200 °C. The GC oven temperature program was programmed to have an initial temperature of 50 °C, and the temperature increased at a rate of 2 °C/min to 260 °C. A 5% *w*/*v* solution of the sample in CH_2_Cl_2_ was prepared, and 0.1 μL was injected with a splitting mode (30:1). Identification of the oil components was based on their retention indices determined by reference to a homologous series of *n*-alkanes, and by comparison of their mass spectral fragmentation patterns with those reported in the literature [22], and stored in our in-house Sat-Set library [23].

### 2.3. Mosquito Larvicidal Assay

Laboratory-reared larvae of *Ae. aegypti* and *Ae. albopictus* were collected from a mosquito colony maintained at the Laboratory of Parasitology and Entomology of Duy Tan University, Da Nang Vietnam. Wild larvae of *Ae. albopictus* and *Culex quinquefasciatus* (Say) were collected from Hoa Khanh Nam district (16°3′14.9″ N, 108°9′31.2″ E). For the assay, aliquots of the aerial parts (leaves and stems) and essential oils of *E. hieraciifolius* and *E. valerianifolius* (Quang Nam stems & leaves) dissolved in dimethylsulfoxide (DMSO) (1% stock solution of essential oil in DMSO) were placed in 500 mL beakers and added to water that contained 25 larvae (fourth instar). With each experiment, a set of controls using DMSO was also run for comparison. Mortality was recorded after 24 h and again after 48 h of exposure, during which no nutritional supplement was added. The experiments were carried out at 25 ± 2 °C. Each test was conducted with four replicates with six concentrations (100, 80, 50, 25, 12.5, and 5 μg/mL). Permethrin was used as a positive control.

### 2.4. Non-Target Lethality Assays

For the assay against *Daphnia magna* Straus (Cladocera: Daphniiidae), aliquots of the essential oil of *E. hieraciifolius* and *E. valerianifolius* (Quang Nam stems and leaves), dissolved in DMSO (1% stock solution), were placed in 250 mL beakers and added to water that contained 20 larvae (fourth instar). Mortality was recorded after 24 h and 48 h of exposure, during which no nutritional supplement was added. The experiments were carried out at 25 ± 2 °C. Each test was conducted with four replicates with five concentrations (12, 6, 3, 1.5, and 0.75 μg/mL). The assay against *Chiromonus tentans* Fabricius (Diptera: Chironomidae) larvae was carried out as above using four replicates with five concentrations (100, 50, 25, 12.5, and 6 μg/mL). For the assay against *Danio rerio* Hamilton (Cypriniformes: Cyprinidae), young, immature fish around 2–3 cm in size were selected for the experiment. Twenty fish were separated in 2.5 L plastic containers with 1.0 L of tap water, with a temperature of 25 ± 2 °C and external relative humidity of 85%. For each dose (100, 50, 25, 12.5, and 6 μg/mL), four repetitions of the experiment were performed. The mortality of organism non-target was calculated following an exposure period of 24 h. With each experiment, a set of controls using DMSO was also run for comparison.

### 2.5. Data Analysis

The mortalities were recorded 24 h and 48 h after treatment. The data obtained were subjected to log-probit analysis [24] to obtain LC_50_ values, LC_90_ values, 95% confidence limits, and chi square values using Minitab^®^ 18 (Minitab Inc., State College, PA, USA). For comparison, LC_50_ values were also determined using the Reed–Muench method [25].

## 3. Results and Discussion

The essential oils from the aerial parts of *E. valerianifolius* and *E. hieraciifolius* were obtained in 1.53 and 1.47% yields, respectively.

### 3.1. Essential Oil Compositions

The chemical compositions of the essential oil of *E. hieraciifolius* and *E. valerianifolius* are presented in Table 1 and Table 2, respectively. The essential oil from the aerial parts (leaves and stems) of *E. hieraciifolius* was rich in the monoterpene hydrocarbons α-pinene (14.5%) and limonene (21.4%), as well as the oxygenated sesquiterpenoid caryophyllene oxide (15.1%). The floral essential oil of *E. hieraciifolius* was also rich in α-pinene (11.8%) and limonene (29.8%), but β-caryophyllene (22.1%) was the dominant sesquiterpene.

The essential oil from the aerial parts (stems and leaves) of *E. valerianifolius* was dominated by the monoterpene hydrocarbons myrcene (47.8%) and α-pinene (30.2%), with a lesser quantity of the sesquiterpene β-caryophyllene (5.4%) (Table 2). The floral essential oils of *E. valerianifolius* were also rich in myrcene (57.0 and 60.6%) and α-pinene (32.5 and 30.6%).

*Erechtites hieraciifolius* and *E. valerianifolius* essential oils from other geographical locations have shown wide variations in chemical composition (Table 3). Thus, α-phellandrene (41.3%) and *p*-cymene (22.2%) dominated the essential oil of *E. hieraciifolius* from Pacoti-Ceara, Brazil [26], while these compounds were only minor components in the sample from Vietnam. Likewise, dillapiole (33.8%) was the major component in *E. hieraciifolius* from Parana State, Brazil [27]; this compound was not observed in the essential oils from Vietnam. The essential oil compositions of *E. valerianifolius* from Vietnam were qualitatively similar to those reported by do Amaral and co-workers from southern Brazil [27], but with major quantitative differences.

It is not clear why there is so much variation in the essential oils of *Erechtites* species. The phytochemical variations may be due to genetic variation. For example, the Missouri Botanical Garden [28] lists six varieties of *H. hieraciifolius* native to the Americas: var. *cacalioides* (Fisch. Ex Spreng.) Griseb (West Indies, Central and South America), var. *carduifolius* (Cass.) Griseb (West Indies), var. *hieraciifolius* (North America and West Indies), var. *intermedia* Fernald (North America), var. *megalocarpus* (Fernald) Cronquist (North America), and var. *praealtus* (Raf.) Fernald (North America). In addition, climatic and edaphic factors, maturity, and phenology can also be responsible for phytochemical variations, particularly in wide-ranging species. For example, several chemotypes of *Artemisia absinthium* L. (Asteraceae) are known, based largely on geographical location [29]. The essential oil of *Peperomia pelucida* (L.) Kunth (Piperaceae) also shows wide variation depending on the geographical source of material [30].

### 3.2. Mosquito Larvicidal Activities

The essential oils from the aerial parts of *E. hieraciifolius* and *E. valerianifolius* collected from Vietnam were screened for mosquito larvicidal activity (Table 4 and Table 5). Larvicidal activity of permethrin (positive control) is shown in Table 6.

The essential oils from the aerial parts of both *E. hieraciifolius* and *E. valerianifolius* showed excellent larvicidal activity against *Ae. aegypti*. The 24 h LC_50_ values were 10.6 and 12.5 μg/mL, respectively, which compare very favorably with other essential oils reported in the literature against this species [33,34,35]. Similarly, the larvicidal activities for the two *Erechtites* essential oils against *Ae. albopictus* were also very encouraging, with 24 h LC_50_ values of 10.5 and 5.8 μg/mL for *E. hieraciifolius* and *E. valerianifoliu*, respectively. Notably, the laboratory-reared *Ae. albopictus* larvae were more susceptible, based on the 95% confidence limits, to *E. valerianifolius* essential oil than the larvae obtained from the wild (24 h LC_50_ = 42.1 μg/mL). Likewise, wild *Culex quinquefasciatus* showed less susceptibility than the laboratory-reared mosquitoes.

Mosquito larvicidal activities (LC_50_) of essential oils against *Cx. quinquefasciatus* have generally ranged between 25.6 μg/mL and 225 μg/mL [36,37]. Thus, the *Cx. quinquefasciatus* larvicidal activity of *E. valerianifolius* (LC_50_ = 40.65 μg/mL) was good compared to other essential oils.

The major components of *E. hieraciifolius* aerial parts essential oil were α-pinene, limonene, and caryophyllene oxide. Both α-pinene and limonene have shown good larvicidal activities against *Ae. aegypti* and *Ae. albopictus* (see Table 7). The LC_50_ values for (+)-limonene average 35.1 and 29.8 against *Ae. aegypti* and *Ae. albopictus*, respectively. Caryophyllene oxide, however, has not shown good larvicidal activity, with LC_50_ values > 100 μg/mL against all mosquito species reported (Table 7).

The larvicidal activities of *E. hieraciifolius* and *E. valerianifolius* essential oils can be attributed to the high concentrations of α-pinene and limonene in *E. hieraciifolius* oil and α-pinene, myrcene, and β-caryophyllene in *E. valerianifolius* oil. However, synergy between essential oil components may also be important [49,52]. Scalerandi and coworkers have demonstrated that *Musca domestica* preferentially metabolizes the major components in an essential oil while leaving the components of lower concentrations to act as toxicants [53].

In order to assess the potential environmental impact of using *Erechtites* essential oils as a larvicidal control agent, we have carried out lethality assays on non-target aquatic species: the water flea, *Daphnia magna* Straus (Cladocera: Daphniidae); non-biting midge larvae, *Chironomus tentans* Fabricius (Diptera: Chironomidae); and zebrafish, *Danio rerio* Hamilton (Cypriniformes: Cyprinidae) (Table 8).

Unfortunately, the *Erechtites* essential oils also show toxicity to representative non-target organisms, with LC_50_ values against the midge larvae (*C. tentans*) and the zebrafish (*D. rerio*) comparable to those for laboratory-reared mosquito larvae. The small crustacean (*D. magna*) was particularly susceptible to the *Erechtites* essential oils. Therefore, care must be taken if these essential oils are to be used in broad applications. Local application of *Erechtites* essential oils (e.g., urban areas) may prove useful as controls for container-breeding mosquitoes, however.

## 4. Conclusions

*Erechtites hieraciifolius* and *E. valerianifolius* are introduced weedy species that grow prolifically in Vietnam, particularly where forests have been cleared; acquisition of abundant quantities of plant material should not be a problem. Mosquito larvicidal screening of these two species indicates good larvicidal activity, which can be attributed to their major components. Thus, this work provides evidence that otherwise noxious introduced weeds might provide low-cost vector control agents to prevent the spread of arboviral infections in Vietnam.

## Figures and Tables

**Table 1:** Chemical compositions of *Erechtites hieraciifolius* essential oils from Vietnam.

RI	Compound	Area %	
		Leaves & Stems	Flowers
921	Tricyclene	---	tr
924	α-Thujene	---	tr
932	α-Pinene	14.5	11.8
948	Camphene	---	0.1
971	Sabinene	0.6	0.7
976	β-Pinene	0.4	0.4
988	Myrcene	2.7	4.4
1006	α-Phellandrene	---	0.3
1016	α-Terpinene	---	tr
1024	*p*-Cymene	0.4	0.1
1028	Limonene	21.4	29.8
1031	β-Phellandrene	---	0.5
1034	(*Z*)-β-Ocimene	---	1.2
1044	(*E*)-β-Ocimene	---	2.3
1057	γ-Terpinene	---	0.1
1084	Terpinolene	---	0.1
1108	Unidentified	0.8	---
1120	*trans-p*-Mentha-2,8-dien-1-ol	0.8	---
1124	Cycloctanone	0.6	---
1125	α-Campholenal	0.6	---
1127	*allo*-Ocimene	---	tr
1135	*cis-p*-Mentha-2,8-dien-1-ol	0.9	---
1140	*trans*-Pinocarveol	0.7	---
1140	*cis*-Verbenol	0.3	---
1144	*trans*-Verbenol	3.5	---
1179	Terpinen-4-ol	0.4	---
1185	Cryptone	1.4	---
1194	Myrtenol	0.8	---
1197	Dodecane	---	0.1
1198	*cis*-Piperitol	0.8	---
1205	Verbenone	1.4	---
1209	Unidentified	0.5	---
1214	Unidentified	1.1	---
1217	*trans*-Carveol	3.5	---
1225	Unidentified	0.7	---
1230	*cis*-Carveol	1.1	---
1242	Carvone	2.0	---
1270	Unidentified	0.8	---
1284	Bornyl acetate	---	0.2
1287	Limonene dioxide	0.9	---
1297	Tridecane	---	0.2
1309	Unidentified	2.2	---
1317	3-Hydroxycineole	0.4	---
1343	Limonene-1,2-diol	4.7	---
1345	α-Cubebene	---	0.1
1357	Neryl acetate	---	0.1
1367	Cyclosativene	---	0.1
1374	α-Copaene	0.6	1.9
1378	*trans-p*-Menth-6-en-2,8-diol	4.1	---
1386	β-Cubebene	---	0.7
1387	β-Elemene	0.6	3.5
1397	Tetradecane	---	0.2
1402	α-Gurjunene	---	1.1
1419	β-Caryophyllene	3.0	22.1
1450	(*E*)-β-Farnesene	---	2.0
1454	α-Humulene	0.5	1.8
1470	*trans*-Cadina-1(6),4-diene	---	0.1
1472	γ-Gurjunene	---	0.2
1473	γ-Muurolene	---	0.1
1480	Germacrene D	---	2.6
1482	(*Z*,*Z*)-α-Farnesene	---	0.7
1486	Valencene	---	0.7
1488	Viridiflorene	---	0.7
1490	*trans*-Muurola-4(14),5-diene	---	0.3
1494	*epi*-Cubebol	---	0.5
1496	α-Muurolene	---	1.2
1501	(*E*,*E*)-α-Farnesene	---	0.1
1514	Cubebol	---	0.2
1516	δ-Cadinene	---	1.4
1549	Isocaryphyllene oxide	1.2	---
1559	(*E*)-Nerolidol	---	0.3
1582	Caryophyllene oxide	15.1	1.6
1607	Humulene epoxide II	0.9	---
1622	Cyperotundone A	---	0.1
1627	1-*epi*-Cubenol	---	0.2
1637	Caryophylla-4(12),8(13)-dien-5β-ol	0.6	0.1
1641	τ-Cadinol	---	0.4
1643	τ-Muurolol	---	0.2
1644	Cubenol	0.5	---
1646	α-Muurolol (Torreyol)	---	0.1
1654	α-Cadinol	0.7	0.3
1658	Selin-11-en-4α-ol	---	0.1
1667	14-Hydroxy-9-*epi*-(*E*)-caryophyllene	1.4	---
1700	Heptadecane	---	0.2
1831	Neophytadiene	---	0.3
1900	Nonadecane	---	0.2
2103	(*E*)-Phytol	---	0.4
	Monoterpene hydrocarbons	40.0	51.7
	Oxygenated monoterpenoids	28.2	0.3
	Sesquiterpene hydrocarbons	4.7	41.3
	Oxygenated sesquiterpenoids	19.2	4.1
	Others	0.6	1.5
	Total Identified	92.7	99.0

**Table 2:** Chemical compositions of *Erechtites valerianifolius* essential oils from Vietnam.

RI	Compound	Quang Nam	Quang Nam	Da Nang
		Leaves & stems	Flowers	Flowers
922	Tricyclene	tr	tr	tr
924	α-Thujene	tr	0.1	tr
933	α-Pinene	30.2	32.5	30.6
949	Camphene	0.1	0.1	0.1
952	Thuja-2,4(10)-diene	tr	tr	tr
971	Sabinene	0.7	1.0	0.9
977	β-Pinene	0.3	0.4	0.3
990	Myrcene	47.8	57.0	60.6
1006	α-Phellandrene	0.3	tr	tr
1016	α-Terpinene	tr	tr	tr
1024	*p*-Cymene	0.1	tr	tr
1028	Limonene	1.4	2.5	1.5
1030	β-Phellandrene	0.1	0.2	0.2
1034	(*Z*)-β-Ocimene	0.3	0.1	tr
1044	(*E*)-β-Ocimene	1.4	0.4	0.2
1057	γ-Terpinene	0.1	0.1	0.1
1084	Terpinolene	tr	0.1	0.1
1100	Undecane	---	tr	tr
1101	Perillene	0.1	tr	tr
1102	Linalool	tr	tr	tr
1112	(*E*)-4,8-Dimethylnona-1,3,7-triene	tr	tr	tr
1128	α-Campholenal	0.1	---	---
1146	*trans*-Verbenol	---	tr	tr
1181	Terpinen-4-ol	0.1	tr	tr
1229	Thymol methyl ether	tr	---	---
1333	δ-Elemene	0.1	0.1	0.1
1374	α-Copaene	0.1	tr	tr
1380	*cis*-β-Elemene	0.1	tr	tr
1382	β-Bourbonene	tr	tr	tr
1386	β-Cubebene	---	tr	0.3
1387	β-Elemene	2.4	0.2	0.1
1400	Methyl eugenol	tr	---	---
1401	α-Gurjunene	0.1	---	---
1411	Dimethoxy-*p*-cymene	0.2	---	---
1418	β-Caryophyllene	5.4	2.7	2.2
1427	γ-Elemene	0.1	tr	tr
1428	β-Copaene	0.1	tr	tr
1450	(*E*)-β-Farnesene	0.2	tr	tr
1454	α-Humulene	0.7	0.3	0.3
1471	γ-Selinene	0.2	---	---
1473	γ-Muurolene	0.1	tr	tr
1480	Germacrene D	3.3	1.8	1.8
1486	Viridiflorene	0.3	---	---
1488	β-Selinene	0.2	tr	tr
1491	*trans*-Muurola-4(14),5-diene	0.1	tr	tr
1494	α-Selinene	0.4	---	---
1494	Bicyclogermacrene	---	0.1	0.2
1496	α-Muurolene	0.1	0.1	tr
1501	(*E*,*E*)-α-Farnesene	0.7	tr	0.1
1511	γ-Cadinene	tr	tr	tr
1516	δ-Cadinene	0.2	0.1	0.1
1558	Germacrene B	0.1	tr	tr
1576	Spathulenol	0.1	tr	tr
1582	Caryophyllene oxide	0.7	0.1	0.1
1609	Humulene epoxide II	0.1	---	---
1622	Cyperotundone A	0.1	---	---
1627	*iso*-Spathulenol	tr	---	---
1642	τ-Cadinol	0.1	tr	tr
1643	τ-Muurolol	0.1	tr	tr
1655	α-Cadinol	0.1	tr	tr
1659	Selin-11-en-4α-ol	0.1	---	---
1684	Germacra-4(15),5,10(14)-trien-1α-ol	---	---	tr
1700	Heptadecane	0.1	0.1	0.1
1832	Neophytadiene	0.2	---	tr
1900	Nonadecane	---	tr	0.1
1944	α-Springene	0.1	0.1	0.1
2100	Heneicosane	---	tr	tr
	Monoterpene hydrocarbons	82.9	94.3	94.6
	Oxygenated monoterpenoids	0.3	tr	tr
	Sesquiterpene hydrocarbons	14.9	5.4	5.1
	Oxygenated sesquiterpenoids	1.3	0.1	0.1
	Others	0.4	0.1	0.2
	Total Identified	99.9	100.0	100.0

**Table 3:** Major chemical components (>5%) of *Erechtites* essential oils.

*Erechtites* Species	Geographical Location	Major Components	Ref.
*E. hieraciifolius*	Pacoti-Ceara, Brazil	α-phellandrene (41.3%), *p*-cymene (22.2%), β-caryophyllene (7.4%), camphor (5.4%)	[26]
*E. hieraciifolius*	Chimoré area, Chapare Province, Bolivia	α-pinene (48.0%), (*E*)-β-ocimene (13.9%), myrcene (13.7%)	[31]
*E. hieraciifolius*	“Private Reservation of Natural Heritage”, Parana State, Brazil	dillapiole (33.8%), α-pinene (33.0%), β-pinene (14.7%), limonene (9.7%)	[27]
*E. valerianifolius*	Mérida, Venezuela	limonene (56.7%), myrcene (12.7%), (*E*)-β-farnesene (10.2%), α-phellandrene (8.7%)	[32]
*E. valerianifolius*	“Private Reservation of Natural Heritage”, Parana State, Brazil	α-pinene (25.8%), sabinene (17.0%), myrcene (16.7%), β-pinene (13.3%), limonene (12.6%)	[27]

**Table 4:** Mosquito larvicidal activity of *Erechtites hieraciifolius* aerial parts (leaves and stems) essential oil.

Mosquito Species	Treatment Time	LC_50_, μg/Ml ^a^ (Fiducial Limits)	LC_90_, μg/Ml ^a^ (Fiducial Limits)	Regression Equation	χ^2^	*p*
*Ae. Albopictus* ^b^	24 h	10.47 (9.12–11.70) *10.06 ± 0.92*	21.11 (19.28–23.59)	*y* = −1.764 + 0.1443*x*	17.6	< 0.001
*Ae. Albopictus* ^b^	48 h	5.49 (1.99–7.87) *6.50 ± 2.38*	18.64 (15.95–22.92)	*y* = −0.177 + 0.0782*x*	12.68	0.002
*Ae. Aegypti* ^b^	24 h	10.58 (9.42–11.68) *10.43 ± 1.93*	19.47 (17.82–21.76)	*y* = −2.078 + 0.172*x*	14.34	0.001
*Ae. Aegypti* ^b^	48 h	8.83 (7.76–9.79) *8.65 ± 1.56*	16.27 (14.89–18.21)	*y* = −2.073 + 0.206*x*	35.49	< 0.001

^a^ There was no mortality in the dimethylsulfoxide (DMSO) controls; LC_50_ values in italics are from Reed–Muench analysis. ^b^ Laboratory-reared mosquito larvae.

**Table 5:** Mosquito larvicidal activity of *Erechtites valerianifolius* aerial parts (leaves and stems) essential oil.

Mosquito Species	Treatment Time	LC_50_, μg/Ml ^a^ (Fiducial Limits)	LC_90_, μg/Ml ^a^ (Fiducial Limits)	Regression Equation	χ^2^	*p*
*Ae. Albopictus* ^b^	24 h	6.07 (5.44–6.73) *6.38 ± 0.72*	11.10 (10.11-12.42)	*y* = −2.110 + 0.306*x*	1.02	0.599
*Ae. Albopictus* ^b^	48 h	4.65 (4.11–5.25) *5.32 ± 1.11*	9.01 (7.96–10.67)	*y* = −1.892 + 0.352*x*	2.26	0.323
*Ae. Albopictus* ^c^	24 h	38.01 (33.56–43.39) *40.71 ± 8.44*	75.84 (65.43–94.11)	*y* = −1.796 + 0.041*x*	5.83	0.016
*Ae. Albopictus* ^c^	48 h	38.57 (34.47–43.73) *35.59 ± 6.58*	67.80 (59.41–81.64)	*y* = −1.691 + 0.044*x*	5.36	0.021
*Ae. Aegypti* ^b^	24 h	12.56 (11.21–13.84) *12.64 ± 2.25*	23.72 (21.78–26.34)	*y* = −1.981 + 0.137*x*	7.69	0.006
*Ae. Aegypti* ^b^	48 h	9.60 (7.97–11.01) *9.40 ± 1.55*	22.22 (20.15–25.07)	*y* = −1.422 + 0.122*x*	22.53	< 0.001
*Cx. Quinquefasciatus* ^c^	24 h	40.06 (37.08–42.64) *40.00 ± 4.92*	55.19 (51.92–59.82)	*y* = −4.316 + 0.101*x*	5 × 10^−7^	0.999
*Cx. Quinquefasciatus* ^c^	48 h	39.48 (36.73–42.23) *37.53 ± 5.26*	53.18 (49.70–58.00)	*y* = −3.697 + 0.094*x*	1.2 × 10^−6^	0.999

^a^ There was no mortality in the DMSO controls; LC_50_ values in italics are from Reed–Muench analysis. ^b^ Laboratory-reared mosquito larvae. ^c^ Wild mosquito larvae.

**Table 6:** Mosquito larvicidal activity of permethrin (positive control).

Mosquito Species	Treatment Time	LC_50_, μg/Ml ^a^ (Fiducial Limits)	LC_90_, μg/mL ^a^ (Fiducial Limits)	Regression Equation	χ^2^	*p*
*Ae. Albopictus* ^b^	24 h	0.0023 (0.0021–0.0026) *0.0022 ± 0.0003*	0.0042 (0.0038–0.0049)	*y* = −1.628 + 686.9*x*	4.73	0.030
*Cx. Quinquefasciatus* ^b^	24 h	0.0167 (0.0152–0.0183) *0.0148 ± 0.0011*	0.0294 (0.0270–0.0326)	*y* = −2.292 + 121.6*x*	26.62	< 0.001

^a^ There was no mortality in the DMSO controls; LC_50_ values in italics are from Reed-Muench analysis. ^b^ Wild mosquito larvae.

**Table 7:** Mosquito larvicidal activities (24 h LC_50_) of essential oil components against various mosquito species.

Compound	Mosquito Species	LC_50_ (μg/mL)	Ref.
β-caryophyllene	*Aedes aegypti*	88.30	[38]
β-caryophyllene	*Aedes aegypti*	38.58	[39]
β-caryophyllene	*Aedes albopictus*	44.77	[40]
β-caryophyllene	*Aedes albopictus*	39.52	[39]
β-caryophyllene	*Anopheles subpictus*	41.66	[40]
β-caryophyllene	*Culex pipiens pallens*	93.65	[38]
β-caryophyllene	*Culex pipiens pallens*	47.79	[39]
β-caryophyllene	*Culex tritaeniorhynchus*	48.17	[40]
β-caryophyllene	*Ochlerotatus togoi*	97.90	[38]
caryophyllene oxide	*Aedes aegypti*	125	[41]
caryophyllene oxide	*Aedes aegypti*	113.00	[39]
caryophyllene oxide	*Aedes albopictus*	107.62	[39]
caryophyllene oxide	*Culex pipiens pallens*	126.28	[39]
limonene	*Aedes aegypti*	19.4	[42]
limonene	*Aedes aegypti*	18.1	[43]
limonene	*Aedes albopictus*	15.0	[42]
limonene	*Aedes albopictus*	32.7	[43]
(+)-limonene	*Aedes aegypti*	27	[44]
(+)-limonene	*Aedes aegypti*	24.47	[38]
(+)-limonene	*Aedes aegypti*	71.9	[45]
(+)-limonene	*Aedes aegypti*	37	[41]
(+)-limonene	*Aedes aegypti*	15.31	[39]
(+)-limonene	*Aedes albopictus*	35.99	[46]
(+)-limonene	*Aedes albopictus*	41.2	[45]
(+)-limonene	*Aedes albopictus*	10.77	[39]
(+)-limonene	*Aedes albopictus*	19.15	[47]
(+)-limonene	*Aedes albopictus*	41.75	[48]
(+)-limonene	*Culex pipiens pallens*	13.26	[38]
(+)-limonene	*Culex pipiens pallens*	10.76	[39]
(+)-limonene	*Culex quinquefasciatus*	40	[49]
(+)-limonene	*Ochlerotatus togoi*	19.20	[38]
(-)-limonene	*Aedes aegypti*	30	[44]
(-)-limonene	*Aedes albopictus*	34.89	[46]
(-)-limonene	*Aedes albopictus*	15.01	[47]
myrcene	*Aedes aegypti*	35.8	[43]
myrcene	*Aedes aegypti*	27.9	[42]
myrcene	*Aedes aegypti*	66.42	[38]
myrcene	*Aedes aegypti*	39.51	[39]
myrcene	*Aedes albopictus*	27.0	[43]
myrcene	*Aedes albopictus*	23.5	[42]
myrcene	*Aedes albopictus*	35.98	[39]
myrcene	*Aedes albopictus*	37.76	[47]
myrcene	*Culex pipiens pallens*	66.28	[38]
myrcene	*Culex pipiens pallens*	41.31	[39]
myrcene	*Culex quinquefasciatus*	167	[49]
myrcene	*Ochlerotatus togoi*	64.76	[38]
α-pinene	*Aedes aegypti*	15.4	[50]
α-pinene	*Aedes aegypti*	79.1	[43]
α-pinene	*Aedes albopictus*	74.0	[43]
α-pinene	*Aedes albopictus*	34.09	[40]
α-pinene	*Anopheles subpictus*	32.09	[40]
α-pinene	*Culex quinquefasciatus*	95	[49]
α-pinene	*Culex tritaeniorhynchus*	36.75	[40]
(+)-α-pinene	*Aedes aegypti*	50.92	[38]
(+)-α-pinene	*Aedes aegypti*	51.28	[39]
(+)-α-pinene	*Aedes albopictus*	68.68	[46]
(+)-α-pinene	*Aedes albopictus*	55.65	[39]
(+)-α-pinene	*Culex pipiens molestus*	47	[51]
(+)-α-pinene	*Culex pipiens pallens*	53.96	[38]
(+)-α-pinene	*Culex pipiens pallens*	60.84	[39]
(+)-α-pinene	*Ochlerotatus togoi*	47.25	[38]
(-)-α-pinene	*Aedes aegypti*	64.80	[38]
(-)-α-pinene	*Aedes aegypti*	39.98	[39]
(-)-α-pinene	*Aedes albopictus*	72.30	[46]
(-)-α-pinene	*Aedes albopictus*	28.61	[39]
(-)-α-pinene	*Culex pipiens molestus*	49	[51]
(-)-α-pinene	*Culex pipiens pallens*	70.36	[38]
(-)-α-pinene	*Culex pipiens pallens*	31.98	[39]
(-)-α-pinene	*Ochlerotatus togoi*	57.93	[38]

**Table 8:** Non-target lethality (LC_50_, μg/mL) of *Erechtites hieraciifolius* and *Erechtites valerianifolius* aerial parts (leaves and stems) essential oils.

*Erechtites hieraciifolius*						
Non-Target Species	Treatment Time	LC_50_, μg/Ml ^a^ (Fiducial Limits)	LC_90_, μg/mL^a^ (Fiducial Limits))	Regression Equation	χ^2^	*P*
*Daphnia magna*	24 h	0.931 (0.808–1.035) *0.909 ± 0.169*	1.531 (1.386–1.767)	*y* = −1.897 + 0.153*x*	8.2 × 10^−4^	0.977
*Daphnia magna*	48 h	0.874 (0.754–0.974) *0.864 ± 0.180*	1.431 (1.297–1.644)	*y* = −2.011 + 2.301*x*	8.1 × 10^−5^	0.993
*Chironomus tentans*	24 h	10.01 (9.18–10.90) *9.37 ± 0.57*	14.73 (13.46–16.71)	*y* = −2.723 + 0.272*x*	0.0037	0.951
*Chironomus tentans*	48 h	7.81 (6.27–9.03) *7.64 ± 0.51*	15.42 (13.56–18.73)	*y* = −1.315 + 0.168*x*	0.370	0.543
*Danio rerio*	24 h	12.41 (11.11–13.78) *11.21 ± 1.47*	21.18 (19.12–24.22)	*y* = −1.897 + 0.153*x*	1.34	0.247
*Daphnia magna*	24 h	0.969 (0.871–1.061) *0.937 ± 0.150*	1.471 (1.347–1.656)	*y* = −2.478 + 2.556*x*	1.7 × 10^−5^	0.997
*Daphnia magna*	48 h	0.917 (0.837–0.999) *0.901 ± 0.119*	1.298 (1.190–1.464)	*y* = −3.081 + 3.361*x*	0	1.0
*Chironomus tentans*	24 h	10.12 (8.85–11.40) *10.08 ± 2.58*	17.99 (15.97–21.28)	*y* = −1.650 + 0.163*x*	1.98	0.159
*Chironomus tentans*	48 h	5.63 (2.67–7.47) *6.67 ± 0.81*	16.31 (14.07–20.35)	*y* = −0.677 + 0.120*x*	2.90	0.088
*Danio rerio*	24 h	18.37 (16.89–20.00) *16.75 ± 1.81*	27.77 (25.45–31.04)	*y* = −2.505 + 0.136*x*	11.38	0.001

^a^ There was no mortality in the DMSO controls; LC_50_ values in italics are from Reed–Muench analysis.
